# Scoping Review of Deep Learning Techniques for Diagnosis, Drug Discovery, and Vaccine Development in Leishmaniasis

**DOI:** 10.1155/2024/6621199

**Published:** 2024-01-17

**Authors:** Alireza Sadeghi, Mahdieh Sadeghi, Mahdi Fakhar, Zakaria Zakariaei, Mohammadreza Sadeghi

**Affiliations:** ^1^Intelligent Mobile Robot Lab (IMRL), Department of Mechatronics Engineering, Faculty of New Sciences and Technologies, University of Tehran, Tehran, Iran; ^2^Student Research Committee, Mazandaran University of Medical Sciences, Sari, Iran; ^3^Toxoplasmosis Research Center, Iranian National Registry Center for Lophomoniasis and Toxoplasmosis, Imam Khomeini Hospital, Mazandaran University of Medical Sciences, Sari, Iran; ^4^Toxicology and Forensic Medicine Division, Mazandaran Registry Center for Opioids Poisoning, Antimicrobial Resistance Research Center, Imam Khomeini Hospital, Mazandaran University of Medical Sciences, Sari, Iran; ^5^Student Research Committee, Sari Branch, Islamic Azad University, Sari, Iran

## Abstract

*Leishmania*, a single-cell parasite prevalent in tropical and subtropical regions worldwide, can cause varying degrees of leishmaniasis, ranging from self-limiting skin lesions to potentially fatal visceral complications. As such, the parasite has been the subject of much interest in the scientific community. In recent years, advances in diagnostic techniques such as flow cytometry, molecular biology, proteomics, and nanodiagnosis have contributed to progress in the diagnosis of this deadly disease. Additionally, the emergence of artificial intelligence (AI), including its subbranches such as machine learning and deep learning, has revolutionized the field of medicine. The high accuracy of AI and its potential to reduce human and laboratory errors make it an especially promising tool in diagnosis and treatment. Despite the promising potential of deep learning in the medical field, there has been no review study on the applications of this technology in the context of leishmaniasis. To address this gap, we provide a scoping review of deep learning methods in the diagnosis of the disease, drug discovery, and vaccine development. In conducting a thorough search of available literature, we analyzed articles in detail that used deep learning methods for various aspects of the disease, including diagnosis, drug discovery, vaccine development, and related proteins. Each study was individually analyzed, and the methodology and results were presented. As the first and only review study on this topic, this paper serves as a quick and comprehensive resource and guide for the future research in this field.

## 1. Background

Leishmaniasis is a neglected parasitic disease caused by different species of the *Leishmania* parasite and is endemic in over 90 countries in tropical and subtropical regions worldwide. *Leishmania* is a single-celled parasite that exists in two forms: a motile form (promastigote) found in the vector's body (female *Phlebotomus* mosquitoes) and a nonmotile form (amastigote) that resides in the host's body, usually in infected humans or domestic or wild animals [1, 2]. Different species of *Leishmania* can cause a variety of clinical manifestations with a wide range of severity. Mild forms of the disease typically present as self-resolving skin lesions, while more advanced forms can lead to life-threatening visceral involvement, often affecting the spleen, liver, and bone marrow [3, 4]. In general, clinical classification, leishmaniasis is divided into four main categories: cutaneous leishmaniasis (CL, it can be seen locally or diffusely), mucocutaneous leishmaniasis (MCL), visceral leishmaniasis (VL), and post-kala-azar dermal leishmaniasis (PKDL) [5]. In spite of the recent advancements in diagnostic techniques for leishmaniasis [6], traditional methods still remain the primary approach for diagnosis. These methods can be broadly classified into three subgroups all of which are based on microscopic examination of stained tissue or parasite cultures [7, 8]. The treatment of this disease comprises of antimonials, amphotericin B, miltefosine, paromomycin, pentamidine; and a combination of these medications used together [9–12]. However, the existing drug regimens are old-fashioned and have certain disadvantages, including cytotoxicity, resistance, and the need for more effective, and less toxic alternatives. It is important to urgently develop novel drug regimens that offer improved efficacy and reduced toxicity [12]. Fortunately, advancements in artificial intelligence (AI) and machine learning have the potential to revolutionize the field of leishmaniasis drug discovery. These technologies can help in analyzing complex data sets, and predict potential drug candidates. By reducing human error and providing a more targeted and efficient strategy, AI can accelerate the process of finding effective treatments for leishmaniasis [13]. It is important to note that, our understanding of leishmanial pathogenesis is still limited and incomplete. Despite numerous efforts, the development of a prophylactic vaccine for leishmaniasis remains a challenging task. However, the use of AI/deep learning algorithms has led to the development of various analytical approaches, including computational/in-silico based tools. These tools can specifically design the immunoinformatic based multiepitope vaccine, which offer innovative vaccine candidates [14].

Early diagnosis and prompt treatment are crucial for controlling leishmaniasis [15], but repeated failures in conventional diagnostic methods have led to delays in starting chemotherapy and increased mortality in endemic areas. On the other hand, nowadays deep learning techniques [16, 17], a category of methods that have the capability to recognize intricate patterns in vast datasets, have shown the state-of-the-art performance in many medical applications [18–20]. Compared to the traditional machine learning methods, deep learning is more precise and flexible [17]. For example, many machine learning techniques are limited in processing raw natural data, such as images or voice signals, which require the use of a feature extractor approach in the first step [21]. Although several studies have investigated the application of deep learning methods to protozoan parasites [22–25], to the best of our knowledge, no review studies have been conducted on the implementation of deep learning methods for the *Leishmania* parasite in different domains. Therefore, this paper presents a scoping review of studies that have explored deep learning methods in the aforementioned field.

## 2. Methodology

In this review, a meticulous methodology was employed to identify and select relevant articles pertaining to the application of deep learning techniques in *Leishmania* research. The process involved searching two prominent databases, Google Scholar, Scopus, and PubMed, to gather articles published from 2018 to the present. The aim was to encompass the latest advancements in the intersection of *Leishmania* and deep learning. Specific keywords and their combinations were utilized in the search to ensure precision: “deep learning and *Leishmania*,” “*Leishmania* and image classification,” “*Leishmania* and image segmentation,” “deep learning and leishmania examination,” “deep learning and *Leishmania* protein,” and “deep learning and *Leishmania* diagnosis”.

To maintain relevance, articles were included based on the defined criteria: First, only articles that explicitly employed deep learning techniques were considered. Studies that utilized general machine learning techniques without a deep learning focus were excluded. Moreover, articles written exclusively in English were considered for inclusion. Finally, articles had to directly relate to the interface of *Leishmania* and deep learning, encompassing image classification, image segmentation, examination, protein analysis, and diagnosis. Articles not meeting these criteria were excluded from the review, particularly those using nondeep learning machine learning techniques or published in languages other than English.

## 3. Telemedicine and Deep Learning

Telemedicine has become an increasingly important field of healthcare in recent years. By utilizing technology such as video conferencing, remote monitoring devices, and mobile apps, telemedicine provides patients with access to medical care from anywhere, at any time. Telemedicine has the potential to improve healthcare access for patients who live in remote or underserved areas, as well as those who have difficulty accessing traditional healthcare services due to mobility issues or other disabilities [26].

One of the most promising areas of development in telemedicine is the use of deep learning techniques [16, 17]. Deep learning is a subfield of AI that utilizes neural networks to analyze vast amounts of data and identify patterns. In the context of telemedicine, deep learning can be used to analyze medical images, patient data, and other types of medical information to improve diagnosis and treatment. For example, deep learning algorithms can be trained to identify early signs of diseases such as cancer [27] or diabetes [28], which can help doctors to provide early intervention and improve patient outcomes.

Another area where deep learning can be beneficial in telemedicine is in remote patient monitoring. With the use of wearable devices and other remote monitoring tools, patients can transmit data to their healthcare providers in real-time, allowing for more effective monitoring of their condition. Deep learning algorithms can be used to analyze this data, identifying patterns that may be indicative of health issues before they become serious. This can help healthcare providers to intervene earlier and provide more effective treatment.

Despite the many potential benefits of deep learning in telemedicine, there are also some challenges that must be overcome. One of the biggest challenges is the need for large amounts of high-quality data to train the algorithms [14]. Additionally, there are concerns around the ethical implications of using deep learning to make medical decisions [29], and the potential for bias in the algorithms [30, 31].

In the following paragraphs, we will explore several specific deep learning models that have been applied in the field of leishmaniasis research. These models have been developed to address specific challenges of detecting, monitoring, and predicting the occurrence of this parasitic disease. As we will see, the application of deep learning models in leishmaniasis research has shown immense promise in improving disease management and control.

### 3.1. Artificial Neural Networks (ANN)

The concept of an ANN was first proposed by McCulloch and Pitts [32] in 1943. It is a model structure that consists of a set of fully connected layers. The inputs are first fed into an ANN, and after passing through several hidden layers, the output is generated in the last layer. The model then adjusts itself by comparing the generated output with the ground-truth value, aiming to improve its performance. When an ANN contains many hidden layers, it is referred to as a deep neural network (DNN) [17].

### 3.2. Convolutional Neural Networks (CNNs)

CNN is a widely used neural network model in various applications. Unlike the DNN, in which all neurons in a given layer are connected to all neurons in the adjoining layers, every layer in a CNN model only focuses on specific parts of the previous layer's outputs to extract useful features at each step [33]. This makes CNNs computationally efficient and less time-consuming.

### 3.3. VGGNet

VGGNet [34], proposed by researchers from the Visual Geometry Group at Oxford University, is a simple arrangement of several convolutional, pooling, and dense layers that won the ImageNet Large-Scale Visual Recognition Challenge (ILSVRC) in 2014 [35]. The VGGNet16 model, which contains 16 layers, has become a widely used model in deep learning applications.

### 3.4. ResNet

During the training of a CNN, as the model becomes deeper, some of the information may lose its impact on the final output. In order to address this issue, He et al. [36] proposed the use of skip connections for DNNs, and their model won the ILSVRC competition in 2015. According to their method, skip connections add information from a specific layer to the output of a few layers ahead. ResNet models are typically denoted by a number that indicates the number of convolutional and dense layers used in the model. For example, ResNet34 has 34 of these layers.

### 3.5. U-Net

The U-Net model, proposed by Ronneberger et al. [37], is a widely used architecture for image segmentation tasks, specifically in assigning every pixel of an image to a particular class. The U-Net model consists of two main steps: downsampling and upsampling. In the downsampling step, the model extracts feature from the image, while in the upsampling step, a pixel-wise label map is generated using the extracted features. The architecture of the U-Net model is named after its shape, which resembles the letter “U”.

### 3.6. Generative Adversarial Network (GAN)

**Figure 1:**
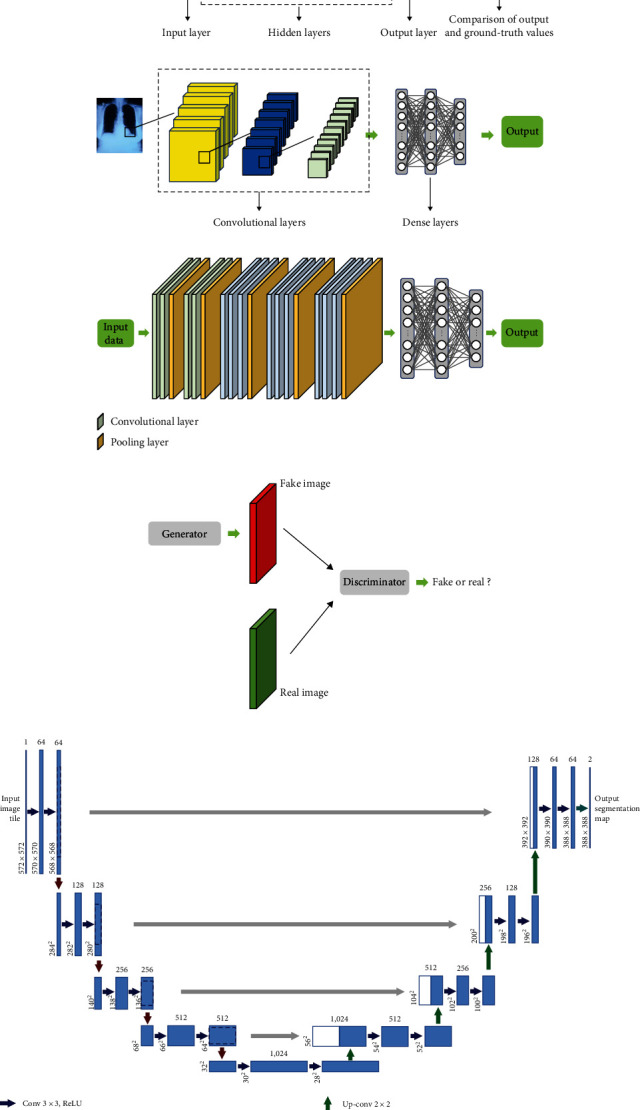
Models' structures: (a) a simplified structure of a typical ANN, (b) simplified structure of a CNN model (c) VGGNet16, (d) GAN structure, and (e) U-Net architecture.

The GAN was first proposed by Goodfellow et al. [38], consisting of two main components: the generator and the discriminator. The primary goal of the GAN is to train the generator to generate new images similar to real images, while simultaneously training the discriminator to distinguish fake images from real images as accurately as possible. Through this conflict between the two units, the model can produce new fake images that are nearly indistinguishable from the real images in the dataset.  Figure 1 illusterates the deep learning models described above.

### 3.7. Transfer Learning

In the context of deep learning, the size and quality of data used to train the model have a significant impact on its ability to generalize and perform well on new data [39]. However, in medical studies, it can be challenging to obtain sufficient amounts of high-quality data due to several factors such as privacy regulations, ethical considerations, and the complexity of medical data. As a result, medical datasets are often limited in size, making it difficult to train deep learning models for the clinical applications [40]. To address this challenge, researchers have turned to an alternative approach known as transfer learning. Transfer learning involves pretraining a deep learning model on large datasets from other domains and then fine-tuning it on the smaller medical dataset. By leveraging the features learned from the large datasets, transfer learning can help to improve the generalization and performance of the model on the smaller medical datasets [41].

### 3.8. Assessments

A deep learning model's performance can be evaluated using various metrics, depending on the objective of the model. Typically, these models are created to address one or more specific problems, such as classification, segmentation, regression, or generation. Each of these problems has its own set of metrics to evaluate model performance.

One of the most common metrics used to evaluate deep learning models is accuracy. Accuracy is the ratio of correctly predicted samples to the total number of samples. However, accuracy alone is not always the most appropriate metric, particularly in cases where the data are imbalanced [42]. In such cases, a model with a high level of accuracy may still perform poorly on the minority class, which is typically of the greatest interest in many applications.

To overcome this issue, a number of alternative metrics have been developed. One such metric is precision. Precision is the ratio of true positives (TP) to the sum of true positives and false positives (FP). Essentially, it measures the percentage of positive predictions that are actually true positives. This metric is particularly useful in cases where false positives are more problematic than the false negatives.

Another metric that is commonly used is recall, also known as sensitivity. Recall is the ratio of true positives to the sum of true positives and false negatives (FN). This metric measures the percentage of true positives that the model was able to correctly identify.

In addition to the metrics mentioned above, specificity is another important performance metric that is sometimes used in deep learning models. Specificity is the ratio of true negatives (TN) to the sum of true negatives and false positives. Essentially, it measures the percentage of negative predictions that are actually true negatives. Similar to recall or sensitivity, specificity is a measure that is useful in specific problem domains, particularly in medical diagnosis or fraud detection where accurate negative predictions are important.

F1-score is another popular metric that combines precision and recall, providing a more balanced measure of a model's performance. Specifically, F1-score is the harmonic mean of precision and recall. Other metrics used for specific tasks include mean-squared error and root-mean-squared error for regression tasks, and Dice score for the segmentation tasks.

**Figure 2:**
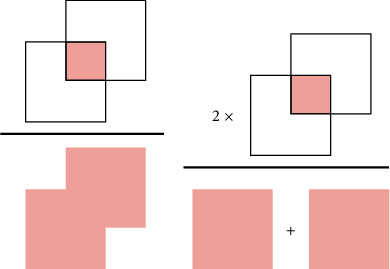
A schematic representation of two commonly used metrics, the Jaccard index (a) and Dice score (b), for evaluating the performance of image segmentation algorithms.

The Dice score and Jaccard index represent two widely employed metrics within the domain of image segmentation tasks. These metrics serve to quantify the efficacy of a deep learning model in delineating and accurately segmenting objects within an image. This concept is further explained in Figure 2.

(1) $$\begin{array}{c} \text{Accuracy}=\frac{\text{TP}+\text{TN}}{\text{TP}+\text{TN}+\text{FP}+\text{FN}}, \end{array}$$

(2) $$\begin{array}{c} \text{Precision}=\frac{\text{TP}}{\text{TP}+\text{FP}}, \end{array}$$

(3) $$\begin{array}{c} \text{Recall or Sensitivity}=\frac{\text{TP}}{\text{TP}+\text{FN}}, \end{array}$$

(4) $$\begin{array}{c} \text{Specificity}=\frac{\text{TN}}{\text{TN}+\text{FP}}, \end{array}$$

(5) $$\begin{array}{c} F1-\text{Score}=\frac{2\times \text{Precision}\times \text{Recall}}{\text{Precision}+\text{Recall}}, \end{array}$$

(6) $$\begin{array}{c} \text{Dice Score}=\frac{2\times \text{Area of Overlap}}{\text{Combined Area}}, \end{array}$$

(7) $$\begin{array}{c} \text{Jaccard index}=\frac{\text{Area of Overlap}}{\text{Area of Union}}. \end{array}$$

Among the deep learning models delineated earlier, CNNs have gained substantial prominence within diverse medical applications, encompassing diagnoses, vaccine development, and drug detection. Their innate capacity to autonomously acquire hierarchical features from images renders them exceedingly versatile for a myriad of undertakings within the medical realm. VGGNet and ResNet, both stemming from the CNN paradigm, have found predominant utility in the domain of medical diagnosis. Notably, UNet's architectural configuration renders it particularly well-suited for tasks involving the segmentation of medical images—wherein precise delineation of regions of interest, such as tumors or anatomical structures, assumes pivotal significance. GANs have garnered widespread employment in medical contexts, primarily for the generation or manipulation of medical images. For example, GANs can be effectively harnessed for denoising medical images. A summary of the applications of these techniques within the medical domain is presented in Table 1.

## 4. Leishmaniasis Diagnosis

In the field of leishmaniasis diagnosis, various conventional (microscopic examination, culture, and serological tests) and novel diagnostic (molecular-based approaches) techniques are used for the detection and diagnosis of leishmaniasis, however, microscopic examination is widely accepted as gold standard. On the other hand, molecular-based techniques can overcome on several limitations of the conventional methods. In this regard, microscopic examination is widely used due to its low cost and simplicity [70, 71]. However, this method is time-consuming and prone to the human errors [72]. On the other hand, deep learning has shown outstanding performance in various image-related applications such as image classification [73, 74], object detection [75, 76], and image segmentation [77, 78]. Therefore, deep learning can be considered as an alternative method for quicker and more accurate diagnosis of leishmaniasis from the microscopic images. The method is a smart tool for identifying *Leishmania* amastigotes (known as Leishman-Donovan bodies) on stained microscopic slides, including archival ones, which can be achieved without the need for experienced personnel and without the need for tools, equipment as well as physical space. Furthermore, this can be done remotely and in field conditions, while some conventional and novel techniques will not have such advantages.  Table 2 summarizes the studies conducted on *Leishmania* diagnosis and their respective particulars.

One of the first studies to employ deep learning as a tool for microscopic examination was presented in [79]. The study aimed to classify various microscopic objects into different categories, with the help of a two-step method. In the first step, the authors employed the popular U-Net model [37] for image segmentation, the process of partitioning an image into multiple segments or regions that are relevant to specific objects or features of interest. Using the segmented images, the authors then proceeded to classify objects into six categories. The categories included background, cytoplasm, nucleus, promastigote, adhered, and amastigote. This classification was achieved through deep learning algorithms that considered various features of the segmented image.

The authors used a total of 45 microscopic images that were manually annotated by experts using a specific tool developed for this task. These images were provided by the Computational Biology and Complex Systems Group at Universitat Politecnica de Catalunya. While this study provided valuable insights into the use of deep learning for microscopic examination, the authors suggested that the model's performance could be improved by using more images to train it and implementing a more accurate method for annotation. This highlights the importance of accurate annotation in deep learning, which is critical to the performance of the model.

The study presented in [80], introduces a model called Cell Explorer, which can identify and count three types of parasites in the microscopic images. The parasites include *Leishmania*, *Trypanosome*, and *Plasmodium*, and the model can identify more than one parasite in a single image, making it a multiple-label model.

The model was trained and implemented using 401 microscopic images obtained from the blood samples of 15 mice, with five mice infected with one of the three parasites. To identify the parasites, the images were preprocessed and a pretrained ResNet18 model [81] was used.

Apart from identifying parasites, the model was also able to count the number of cells present in the microscopic images. This was achieved using the simple linear interactive clustering algorithm [81]. The Cell Explorer model achieved an impressive accuracy of about 95%, with an F1-score of 0.766.

The study in [82], aimed to address the ongoing challenge of accurately detecting cutaneous leishmaniasis (CL) from skin lesion images using a transfer learning approach with VGG19 [34] as the base model in a mobile application. The dataset used in the development of the model comprised 2022 images representing diverse categories, including CL images, melanoma, and a range of other diseases that could potentially be mistaken for CL during diagnosis. The dataset was carefully selected to simulate real-world conditions, where misdiagnosis can occur frequently and impede appropriate medical intervention, ultimately exacerbating the negative clinical outcomes of CL.

Additionally, the study assessed the model's performance against the skills of human experts by requesting seven CL experts to classify 100 random images. The results demonstrated the superiority of the model, recording an impressive accuracy rate of 99%, relative to 83% for the human experts. These results indicate that the developed mobile application has the potential to offer significant support to healthcare practitioners in achieving improved diagnoses, particularly within regions where CL is endemic and medical resources may be limited.

In [83], an automated imaging platform called Octopi was introduced for the diagnosis of parasitic diseases from blood smear. The platform is highly modular and can diagnose different parasitic diseases by utilizing different modules. Octopi is capable of identifying *Plasmodium falciparum*, *Leishmania donovani*, *Trypanosoma brucei rhodesiense*, and several other parasites. Additionally, the platform can count the number of red blood cells, which can be useful for identifying the presence of parasites in infectious blood. To achieve this, Octopi implemented a 91-layer fully convolutional DenseNet [84] model for counting the red blood cells. A total of 22,680 images, all automatically annotated, were used to train the model. Moreover, the model was modified to achieve real-time performance.

In the field of leishmaniasis diagnosis, microscopic images can often be blurry or out-of-focus due to defects in instruments or human error, leading to misdiagnosis by the experts [85]. Furthermore, these issues can negatively impact the performance of related research. To address these challenges, a GAN-based model was proposed in [86] that is capable of deblurring and correcting out-of-focus images. This model was trained on two different datasets that were both self-collected and publicly accessible. This study not only enhances the quality of leishmaniasis microscopic imaging, but it also aids further research in deep learning for leishmaniasis, as the provided dataset includes a large number of leishmaniasis microscopic images. To evaluate the proposed model, the authors compared its performance with several similar models and demonstrated that their model outperforms others.

## 5. Leishmaniasis Drug Discovery

Currently available treatments for leishmaniasis are associated with high costs, long treatment periods, and potential side effects, as highlighted in [87]. This presents a significant challenge, particularly in endemic regions where resources are limited. Therefore, there is a pressing need for developing new treatments that are more cost-effective, safe, and efficient.

One promising approach to develop new treatments for leishmaniasis is the repurposing of existing drugs used to treat other diseases [88]. This approach, also referred to as drug repositioning, has been employed successfully for other infectious diseases like SARS-CoV-2 [89, 90]. The concept involves screening all known drugs to identify those that have potential therapeutic effects against leishmaniasis.

In this context, molecular docking plays a crucial role. It is a computational technique that predicts whether two molecules, the receptor and the ligand, can chemically bind. The conventional software for molecular docking includes AutoDock Vina, Rosetta Ligand, and AutoDock 4, which are widely used in the scientific community [91–93]. However, these conventional methods often face limitations, such as the inability to operate in complex biological environments, inefficiency, and imperfect prediction of the binding affinities.

In recent years, deep learning approaches have been applied in different fields and have shown promising results in prediction tasks [94, 95]. These techniques can support drug discovery by predicting molecular interactions between drugs and target proteins, paving the way for drug repurposing for the treatment of various diseases, including leishmaniasis.

Therefore, by combining molecular docking and deep learning techniques, drug repurposing could be accelerated, with the potential to identify novel drugs and optimize existing ones for managing leishmaniasis. This could lead to more cost-effective and efficient treatment options, improving the prognosis and quality of life for patients suffering from leishmaniasis.

In 2020, the Indaba Grand Challenge was conducted and it sought to identify effective combinations of *Leishmania* proteins and small molecules that could be used to develop a new drug for leishmaniasis. The competition produced a dataset consisting of 4,021 ligands (small molecules) and 512 targets (*Leishmania* proteins), which researchers then used to try to find a suitable drug. In an effort to achieve this objective, Dassi et al. [96] employed a hybrid approach that combined deep learning with molecular docking. Specifically, they utilized the DeepPurpose [97] which is a deep learning library to predict drug-target interactions. After screening the entire dataset, the researchers identified 3,400 promising pairs from a pool of over 2 million possible pairs. The next step was to test these pairs using AutoDock Vina, a widely used molecular docking software that can predict how a ligand will bind to a protein. AutoDock Vina was used as a ground truth method to rank the 3,400 promising pairs and the results obtained using this software were then compared to the rankings predicted by the deep learning library. The comparison revealed that although deep learning was unable to rank the pairs accurately, molecular docking was a more reliable method. However, by leveraging the power of deep learning, they were able to significantly reduce the time required for the entire procedure. From a process that initially took 2 months, the researchers were able to conduct it in just 14 hr.

In a similar investigation to that carried out by Dassi et al. [96]; researchers, Smith et al. [98] sought to identify a viable drug for treating leishmaniasis, utilizing two different deep learning frameworks: DeepPurpose [97] and a multiobjective neural network binding affinity prediction model (MONN) [99]. The DeepPurpose model was trained on the BindingDB dataset [100], which comprises more than 2-million binding data, while the MONN model was trained on the PDBBind v2018 dataset [101], which includes over 17,000 protein-ligand complexes. After implementing these two frameworks and comparing their results with the general truth methods of Autodock Vina [93] and PyRosetta docking [102], Lacosamide was identified as a prospective treatment for leishmaniasis. The researchers also represented each molecule as a real-value vector using Mordred [103] and RDKit. By computing a distance matrix among drug candidates, Conivaptan and Midostaurin were identified as other promising drugs that could potentially aid in the treatment of leishmaniasis.

AI can actually be used to evaluate the toxicity parameters of drugs. AI algorithms can analyze large data sets, including chemical structures and biological data, to predict the safety and effectiveness of drugs. For toxicity assessment, AI models can be trained on existing data from various sources, such as databases and published studies, to recognize patterns and predict potential toxic effects of new drugs. This could help identify drug candidates with a lower risk of toxicity early in the drug discovery and development process, thereby reducing the need for extensive animal testing [96–98]. In addition, AI can also be used to evaluate other pharmacological parameters such as drug interactions, absorption, distribution, metabolism, and excretion (ADME) and pharmacokinetic profiles. By analyzing known molecular properties and structure–activity relationships, AI algorithms can help predict these parameters for new drug candidates [104].

Overall, AI can improve the drug development process by providing valuable information on toxicity and pharmacological parameters, enabling more informed decisions and potentially reducing the time and resources required to bring safe and effective drugs to the market.

## 6. Leishmaniasis Vaccine

Leishmaniasis is responsible for a significant number of fatalities every year, particularly in countries located in Africa and the Middle East [2, 105]. Despite the availability of several methods of therapy and drugs, the burden of this disease continues to be a major public health issue. One of the most effective methods of controlling and eradicating infectious diseases is through vaccination. Vaccines help to prevent the transmission of diseases by providing immunity against the pathogen responsible for the disease.

Therefore, the development of an effective and safe vaccine against leishmaniasis is essential in preventing and controlling this disease. Although there have been some successes in developing vaccines to prevent *Leishmania* transmission from dogs to humans, such as Leishmune and CaniLeish, there is currently no licensed vaccine available for humans [106, 107]. Hence, there is an urgent need to develop and test new vaccines for human protection against leishmaniasis.

There is considerable research interest in developing anti-leishmaniasis vaccines, however, the development of an effective vaccine against leishmaniasis presents several challenges, including the complex nature of the parasite's life cycle and the differences in the immune response generated by different *Leishmania* species.

In an effort to address this issue, Saha et al. [14] developed an immunoinformatics-based chimeric vaccine using four different *Leishmania donovani (L. donovani*) proteins: an ATP-dependent zinc metallopeptidase, a histidine secretory acid phosphatase, a rhomboid-like protein, and an amastin-like surface protein (ALSP). The chimera was constructed by linearly arranging the most effective helper T-lymphocytes (HTL), cytotoxic T-lymphocytes (CTL), and B-cells epitopes of the mentioned proteins. The most promising CTL epitopes were identified using NETMHC 4.0, which utilizes an ANN [108], while the most efficient B-cells epitopes for all four *L. donovani* proteins were detected using ABCpred, another ANN model [109]. The secondary structure of the vaccine was produced using the PSIPRED web server, which contains two neural network models [110], while the 3D structure of the proposed vaccine was generated using the RaptorX web server, a deep learning model [111].

Overall, an active area of research in leishmaniasis is multiepitope vaccines. These vaccines are designed to provide broad protection by integrating multiple epitopes from different Leishmania antigens into a single vaccine construct. Here are some of the tools and approaches used to develop vaccine targets against multiepitope leishmaniasis [112–114]:

1. Bioinformatics tools: Various bioinformatics software and databases are used to predict potential epitopes of *Leishmania* antigens. These tools include algorithms such as NetMHC, BepiPred, and Propred, which help in predicting T and B cell epitopes based on a specific criteria, including antigen processing and presentation.
2. Epitope mapping: Experimental methods like peptide microarrays and mass spectrometry are employed to identify potential epitopes by mapping the regions of antigens that are recognized by the immune system. This information is useful for choosing the most effective and consistent epitopes for the development of vaccines.
3. Immunoinformatics: Immunoinformatics combines the fields of immunology and computer science in order to forecast and examine immune responses. Computational techniques, such as those utilized by the Immune Epitope Database (IEDB) and VaxiJen, are employed to predict the immunogenicity and antigenicity of epitopes. These tools assist in the selection of epitopes that have a strong binding affinity to major histocompatibility complex (MHC) molecules, which are capable of triggering immune responses.

The process of developing multiepitope vaccines for leishmaniasis is indeed a dynamic and ongoing effort. Researchers are continuously improving and refining the tools and techniques used for epitope prediction and mapping, as well as exploring novel approaches and technologies to enhance vaccine effectiveness. This iterative approach allows for continuous progress and the potential for breakthroughs in the fight against leishmaniasis.

## 7. Leishmaniasis 3D Protein Structure

Predicting the structure of proteins is a crucial step in understanding their function and behavior in the biological systems. Proteins are involved in virtually all cellular processes, such as metabolism, signaling, and regulation, and their activity is strongly influenced by their three-dimensional structure. Knowing the precise structure of a protein can help researchers to identify potential drug targets, design new therapeutics, and develop treatments for a wide range of diseases. Additionally, predicting the structure of proteins has important implications for fields such as biotechnology and bioengineering, where it can be used to engineer new proteins with the desired properties.

In the field of bioinformatics, predicting the structure of proteins has been a significant challenge. Different approaches have been employed in the past to achieve this, ranging from statistical to deep learning methods [115–117]. Among these, deep learning has shown remarkable success in the recent years, including AlphaFold2 [118] and RoseTTAfold [119]. These models utilize multiple sequence alignments to predict the 3D protein structure accurately. AlphaFold2 was the winner of the Critical Assessment of protein Structure Prediction (CASP) competition (CASP14) [120], demonstrating its ability to accurately predict the structure of proteins.

However, while AlphaFold2 has performed well in predicting the structures of many species, its accuracy in predicting the structure of proteins from species like *Leishmania infantum* has been suboptimal. This issue arises from the underrepresentation of such species in the AlphaFold2 databases. To address this, researchers in [121] have collected additional data from public protein sequence datasets. The data used in this study consisted of 243 genomes and transcriptomes obtained from different sources. Some of the sources included in the dataset were TriTrypDB [122, 123], a comprehensive database of genomic information on trypanosomatid species, NCBI, a leading source of genetic information that incorporates data from multiple sources, sequencing read archive (SRA), a public repository of sequencing data from different studies, transcriptome shotgun assembly (TSA), which provides transcriptomic data to complement genomic information, and Marine Microbial Eukaryotic Transcriptome Sequencing Project (MMETSP), which provides transcriptome data from marine eukaryotic organisms. In particular, 83 genomes were sourced from TriTrypDB, NCBI, and SRA while 160 transcriptomes were obtained from TSA, MMETSP, and NCBI SRA. By combining data from these different sources, the researchers provided a more representative and comprehensive set of protein sequences to the deep learning model, AlphaFold2. This allowed the model to achieve higher performance in predicting protein structures from a broader range of species, including understudied species like *L. infantum*.

To evaluate the resulting improvement in the model's performance, the predicted local distance difference test score (pLDDT) [124] was used. This metric measures the quality of protein structure predictions, where higher scores indicate better quality. The updated AlphaFold2 model demonstrated remarkable improvements in its ability to predict the structure of proteins from species like *L. infantum*.

Overall, the incorporation of additional data sources into deep learning models can enhance their performance in predicting protein structures accurately. Improved models can support drug discovery efforts, ultimately leading to more effective treatments for diseases like leishmaniasis that result from protein dysfunction.

## 8. Conclusion

This scoping review, as a pioneering review study in this field, is an important first step in the development of machine learning and deep learning techniques, a branch of AI, for diagnosing, treating, and preventing leishmaniasis.  Table 3 presents a summary of the studies that have been analyzed within the context of this research. By providing a comprehensive overview of the current state of research, this review can help to guide future studies and accelerate progress in this critical area. Deep learning holds great promise for the future of leishmaniasis research in terms of diagnosis, treatment, and vaccine development. Here are some perspectives on how deep learning can revolutionize these aspects:

1. Improved diagnostic accuracy: This can greatly assist healthcare professionals in making timely and accurate diagnoses and enable rapid initiation of treatment.
2. Drug discovery and reuse: This approach could potentially streamline the drug development process and lead to more efficient and cost-effective treatments for leishmaniasis.
3. Accelerated vaccine development: Deep learning algorithms can identify potential antigen targets for vaccine development. With its ability to recognize subtle patterns and relationships in large data sets, deep learning can accelerate the process of predicting immunogenic antigens, thereby facilitating the development of effective vaccines against leishmaniasis.

A significant hurdle in the advancement of deep learning models for applications within the domain of leishmaniasis revolves around the constraint imposed by the limited size of available datasets. The proficiency and reliability of a deep learning model are dependent upon its exposure to an extensive dataset that encompasses a wide variety of samples. The samples present in the existing datasets often originate from timeframes predating the current period, consequently failing to accurately capture potential shifts in the manifestation of *Leishmania* within microscopic images as they may appear in the contemporary scenarios.

The application of deep learning techniques also merits consideration due to their capacity to effectively process intricate datasets, conduct complex analyses, and assemble insightful information that may be beyond the human comprehension. The incorporation of these two domains has the potential to yield substantial advancements in the realm of *Leishmania* vaccine development, deserving in-depth investigation in subsequent research endeavors. It is imperative to recognize the pivotal role that vaccine development can undertake in the eradication of leishmania, thereby preventing its evolution into a substantial public health concern.

As a whole, despite these challenges, the future perspective of deep learning in leishmaniasis research is immense. Continued research, collaboration, and investment in both data collection and algorithm development will be essential to fully unlock the benefits that deep learning can offer in the diagnosis, treatment, and vaccine development for leishmaniasis.

## Figures and Tables

**Table 1:** Diverse applications of deep learning models in the field of medicine.

Deep learning model	Medical applications
CNN	Diagnosis: Detecting heart arhythmia in ECG [43], diagnosis of epileptic seizure in EEG [44], automatically detect pneumonia in X-ray images [45], screening COVID-19 in chest X-ray images [46], detecting COVID-19 from CT scans [47], and knee osteoarthritis classification in MRI [48] Drug discovery: Target identification and drug repurposing [49], predicting constitutive androstane receptor agonists [50], predicting molecules' effects to find SARS-CoV-2 drugs [51], and Vaccine: Creating a vaccine: SARS-CoV-2 example [52]

VGGNet	Diagnosis: Detecting COVID-19 in chest X-ray [53], classifying the ocular disease in eye image [54], detection of pneumonia from Chest X-Ray [55], early detection of skin cancer [56], and tuberculosis detection in X-Ray Image [57]

ResNet	Diagnosis: Diagnose intracranial hemorrhage in CT Scanning [58], COVID-19 diagnosis from X-ray images [59], diagnosis of knee osteoarthritis [60], and automatic schizophrenia detection from EEG [61]

U-Net	Diagnosis: Segmenting COVID-19 chest CT images [62], brain tumor segmentation and survival prediction [63], liver and lesion segmentation [64], and dental CBCT images segmentation [65]

GAN	Generating structured data in the medical domain [66], low-dose CT denoising [67], data augmentation in breast ultrasound mass classification [68], and ECG denoising framework [69]

**Table 2:** Studies on *Leishmania* diagnosis.

Study	Objective	Model	Dataset	Leishmania type	Performance	Limitations
Górriz et al. [79]	Segmenting *Leishmania* microscopic images and classifying objects in the images	U-Net [37]	45 Microscopic images annotated Manually	Amastigote and promastigote	Amastigote: Dice score of 0.777 and f1-score of 0.777 Promastigote: Dice score of 0.495 and F1-score of 0.491	Small-sized dataset inaccurately annotating images
Mainye et al. [80]	Identifying and counting *Leishmania*, *Trypanosome*, and *Plasmodium* in microscopic images	Cell Explorer (based on ResNet18)	401 Microscopic images from the blood samples of 15 mice	*Leishmania donovani*	Accuracy: 95% F1-score: 0.766	Small-sized dataset mouse samples, not directly generalizable to humans
Arce-Lopera et al. [82]	Designing a mobile application to detect cutaneous leishmaniasis	VGG19 [34]	2,022 Images (containing CL images, melanoma, and other diseases mistaken for CL)	cutaneous leishmaniasis	Accuracy: 99%	—
Li et al. [83]	Introducing an automated imaging platform, for the diagnosis of parasitic diseases from blood smear	Octopi (based on DenseNet [84])	22,680 Images automatically annotated	*Leishmania donovani*	Did not report performance metrics	Not reporting performance
Zhang et al. [86]	Deblurring and correcting out-of-focus microscopic images	GAN-based model	Two self-collected and one publicly accessible datasets	—	Structural similarity: 0.8951	—

**Table 3:** Summary of the reviewed studies.

Study	Objective	Model	Dataset
Górriz et al. [79]	Segmenting *Leishmania* microscopic images and classifying objects in the images	U-Net	45 Microscopic images annotated manually
Mainye et al. [80]	Identifying and counting *Leishmania*, *Trypanosome*, and *Plasmodium* in microscopic images	ResNet18	401 Microscopic images from the blood samples of 15 mice
Arce-Lopera et al. [82]	Designing a mobile application to detect CL	VGG19	2,022 Images (containing CL images, melanoma, and other diseases mistaken for CL)
Li et al. [83]	Introducing Octopi, an automated imaging platform, for the diagnosis of parasitic diseases from blood smear	DenseNet	22,680 Images automatically annotated
Zhang et al. [86]	Deblurring and correcting out-of-focus microscopic images	GAN-based model	Two self-collected and publicly accessible datasets
Dassi et al. [96]	To identify a potential treatment for leishmaniasis	DeepPurpose	A dataset with 4021 small molecules and 512 *Leishmania* proteins
Smith et al. [98]	To identify a potential treatment for leishmaniasis	DeepPurpose and MONN	BindingDB dataset and PDBBind v2018 dataset
Saha et al. [14]	Immunoinformatics-based chimeric vaccine for Leishmaniasis	Neural network platforms (NETMHC 4.0, ABCpred, PSIPRED, RaptorX)	An ATP-dependent zinc metallopeptidase, a histidine secretory acid phosphatase, a rhomboid-like protein, and an amastin-like surface protein (ALSP)
Wheeler and Yurchenko[121]	To increase the accuracy of AlphaFold2 in predicting the *Leishmania infantum* protein structure	—	83 Genomes from TriTrypDB, NCBI, SRA, and 160 transcriptomes from TSA, MMETSP, and NCBI SRA

## Data Availability

The data that support the findings of this study are available from the corresponding author upon reasonable request.
